# Cubic Nanoparticles for Magnetic Hyperthermia: Process Optimization and Potential Industrial Implementation

**DOI:** 10.3390/nano11071652

**Published:** 2021-06-23

**Authors:** Omar Sánchez Sánchez, Teresa Castelo-Grande, Paulo A. Augusto, José M. Compaña, Domingos Barbosa

**Affiliations:** 1 Departamento de Ingeniería Química y Textil, Facultad de Ciencias Químicas, Universidad de Salamanca, Plaza de los Caídos, 1-5, 37008 Salamanca, Spain; jovvicpl@aeiou.pt; 2 LEPABE—Laboratory for Process Engineering, Environment, Biotechnology and Energy, Faculty of Engineering, University of Porto, 4200-465 Porto, Portugal; tcg@fe.up.pt (T.C.-G.); dbarbosa@fe.up.pt (D.B.); 3 Instituto de Biología Molecular y Celular del Cáncer, Campus Miguel de Unamuno, CSIC/Universidad de Salamanca (GIR Citómica), 37007 Salamanca, Spain; 4 Servicio de Difracción de Rayos-X, Universidad de Salamanca, Pza. de Los Caídos s/n, 37008 Salamanca, Spain; jmcompana@usal.es

**Keywords:** magnetic hyperthermia, magnetic nanoparticles, optimization, economic analysis, plant design and process engineering, cubic particles

## Abstract

Cubic nanoparticles are referred to as the best shaped particles for magnetic hyperthermia applications. In this work, the best set of values for obtaining optimized shape and size of magnetic particles (namely: reagents quantities and proportions, type of solvents, temperature, etc.) is determined. A full industrial implementation study is also performed, including production system design and technical and economic viability.

## 1. Introduction

According to the World Health Organization (WHO), cancer is the main cause of death in the world, having been responsible for 9.6 million of deaths in 2018. In the next two decades, the increase in the number of cases is expected to be 60%, according to a new WHO report [1]. Thus, research in oncology is extremely important to develop therapies to treat this disease and increase the expectation and quality of life of patients. Currently, the main treatments are based on surgery, radiotherapy, and chemotherapy. Thus, these techniques have concentrated most of the research and development till now. Nevertheless, the known side effects of these treatments have not been completely eliminated yet. Due to all this, the search for more efficient techniques and more specific action, with fewer unwanted effects, is important in promoting a better quality of life for patients. Various types of complementary therapies exist and are continuously being developed. In particular, the so-called hyperthermia therapies, which take advantage of the fact that tumor cells have a lower thermal tolerance than healthy cells [2]. In fact, hyperthermia is a therapeutic procedure in which tissues/cells are heated above normal physiological ranges (between 41 and 46 °C) in order to kill tumor or sensitize cells to increase the efficiency of standard therapies. 

A more recent modality is magnetic hyperthermia (MHT) [3,4,5,6], where the increase in temperature occurs by applying alternating magnetic fields to a magnetic material with specific characteristics. In fact, the use of Magnetic Nanoparticles (MNPs) in medicine allows to treat hard-to-reach regions of the body. Chemical manipulation at a nanoscale size has conferred the ability to conjugate biomolecules, such as antibodies, with magnetic particles, for a more effective therapy or to achieve a specific goal, making MNPs ideal vehicles for new therapies based on localized and selective heat dissipation. Thus, MNPs can simultaneously combine various therapeutic functionalities, such as drug carriers, contrast agents in magnetic resonance imaging, or magnetic heating agents. Although MHT has been incorporated into clinical practice to treat a relatively wide range of types of cancer (prostate, esophagus, brain, etc.), today, it has shown to be advantageous in the treatment of sarcomas, carcinomas, and lymphomas.

The ability of the magnetic nanoparticles to generate heat when subjected to alternating magnetic fields is the basis of MHT. In fact, when exposed to an alternating magnetic field, MNPs produce heat through two main mechanisms [7]: hysteresis loss and Néel and Brown relaxation loss. Hysteresis losses occur in large MNPs that have multiple domains, while relaxation losses occur mainly in single domain MNPs. 

Magnetite (iron oxide) particles are well-known for their biocompatibility, possible monodispersity and easiness of synthesis [7,8,9]. Besides, mono-domain particles are usually easy to obtain. Shape and size are important characteristics and it has been proven that critical size of magnetite to form multidomain structure is between 76 and 80 nm for cubic-shaped particles and 128 nm for spherical particles [10]. Supermagnetic behavior is known to exist, at least, below 50–20 nm for magnetite particles, depending on their shape and other characteristics [11,12,13]. Besides, SAR values (Specific Absorption Rate) for cubic-shaped particles have been shown to be higher than the ones presented by spherical-shaped particles [14,15]. The correlation between particles microstructure and MHT response has been demonstrated (e.g., [16,17,18]).

The main methods to produce MNPs are coprecipitation, thermal decomposition, microemulsion, sol-gel, hydrothermal synthesis, and synthesis with polyols [8,9]. These methods have been used to prepare particles with an appropriate composition and size distribution, according to the application in mind. For example, for environmental non-specific treatments, coprecipitation is usually the preferred method [8,19,20,21,22,23], while for biomedical applications where monodispersed particles are usually required, less simple methods, like thermal decomposition and hydrothermal, are preferred [8]. A summary of the main methods and their characteristics is presented in Table 1. 

In the case of magnetic hyperthermia, magnetic nanoparticles must present good size distribution, good shape control, and a good SAR generation capability.

Most of the published literature is concerned with laboratorial methods to produce magnetic nanoparticles for hyperthermia applications, but, so far, to the best of our knowledge, none has studied the economic and technological viability of the scale-up of the method and its possible application at large-scale industrial production (which is an important step towards its widespread production and availability, especially after the recent license of a real medical clinical application of magnetic hyperthermia). This will also limit the number of producing methods that may be selected, as the method must be easily scalable (a fundamental characteristic for a production plant).

The present work is concerned with the maximization of the magnetic hyperthermia capabilities of the particles, without increasing the costs to an unbearable level. Thus, the goal is to produce cubic magnetic nanoparticles with sizes lower than the monodomain critical value and, if possible, the supermagnetism critical value. The optimization of the main variables (ramp-up temperature, stirring speed, type of solvent, proportions of raw materials) for the selected method of production is one of the goals. The other goal is to study the economic and technological viability of the production, in order to set the path for future industrial implementation to support clinical widespread applications.

## 2. Materials and Methods

### 2.1. Reagents

In this study was used: iron (III) acetylacetonate (99%) from Sigma Aldrich (St. Louis, MO, USA), oleic acid (90%) from Panreac (Barcelona, Spain), benzyl ether (99%) from Sigma Aldrich (Madrid, Spain), 4-biphenylcarboxylic acid (99%) from Sigma Aldrich (Madrid, Spain), toluene (99.8%) from Panreac (Barcelona, Spain), hexane (99%) from Panreac (Barcelona, Spain), chloroform (99.9%) from Panreac (Barcelona, Spain), and nitrogen (99%) from Air Liquide (Paris, France). All solutions were made with distilled water.

### 2.2. General Experimental Methodology 

#### 2.2.1. Synthesis of Cubic Magnetic Nanoparticles

For the synthesis of the magnetic nanoparticles, the method developed by Kim et al. in 2008 [24] was followed, which gives rise to MNPs with cubic morphology and with sizes close to the critical size of the superparamagnetic state. In a typical synthesis of magnetite nanocubes, 2 mmol of iron acetylacetonate (Fe(acac)_3_)—$\mathrm{Fe}{\left(C_{5}H_{7}O_{2}\right)}_{3}$, 4.5 mmol of oleic acid ($C_{18}H_{34}$), and 52.5 mmol of benzyl ether $-{\;C}_{14}H_{14}O-$(or 2 mmol of 4-biphenylcarboxylic acid $-{\;C}_{13}H_{10}O_{2}$—depending on the size and shape) were used. The initial mixture is first degassed with nitrogen gas. Then, temperature is risen up to 290 °C at a temperature gradient ramp-up of about 20 °C/min, under energetic stirring. Once the final temperature is reached, the reaction remains at 290 °C for 30 min. At the end of the process, the sample is cooled and a mixture of toluene and hexane in a 4:1 volumetric ratio is added, and the sample centrifuged at 1700 rpm. Finally, chloroform is used to clean the particles. The main reaction that occurs at these conditions is:

(1) $$\mathrm{aFe}{\left(C_{5}H_{7}O_{2}\right)}_{3}+{\mathrm{bC}}_{18}H_{34}+{\;\mathrm{cC}}_{13}H_{10}O_{2}/{\mathrm{dC}}_{14}H_{14}O\;\to {\;\mathrm{eFe}}_{3}O_{4}+\mathrm{Subproducts}$$

**Figure 1:**
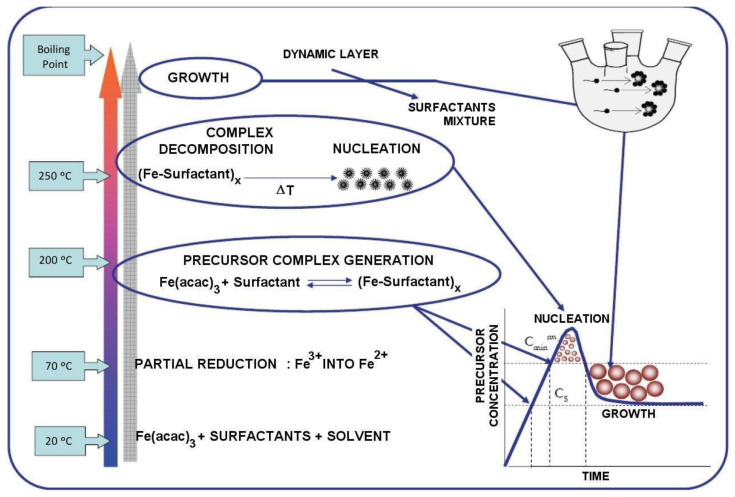
Reaction mechanism—based on [25].

The steps of the reaction process are represented in Figure 1.

#### 2.2.2. Particles’ Characterization

Particles were characterized by determining their magnetic properties, size, and shape and percentage of magnetite. Hence, to certify the percentage of obtained magnetite, X-ray studies were made (X-ray diffraction (XRD) Bruker D8 Advance, Karlsruhe, Germany); to determine the morphology and size of the particles, Scanning Electron Microscopy (SEM) (JEOL JSM-840, Madrid, Spain) was performed; finally, to access magnetic susceptibility, samples were analyzed with a Kappabridge KLY-4 susceptometer (a semiautomatic auto balance inductivity bridge, Porto, Portugal).

#### 2.2.3. Influence of the Main Variables 

For all these experiments, the default options are: iron acetylacetonate (III)—2 mmol; oleic acid—4.5 mmol; 4-biphenylcarboxylic acid—2 mmol; stirring—200 rpm; reaction temperature—290 °C; temperature ramp-up: 20 °C/min; centrifugal speed—1700 rpm. The analyzed variables were:

(a) Effect of mechanical stirring (four values were used: 150, 170, 200, and 220 rpm).
(b) Effect of temperature ramp-up (the increasing temperature gradients studied were: 15, 20, 25, and 30 °C/min).
(c) Effect of the type of solvent (the solvents studied were: (a) 4-biphenylcarboxylic acid—2 mmol; (b) benzyl ether—52.5 mmol; (c) both solvents: 4-biphenylcarboxylic acid—2 mmol—and benzyl ether—52.5 mmol).
(d) Effect of reagents ratio (the ratios studied were: doubling oleic acid amount, doubling 4-biphenylcarboxylic acid amount, and doubling all reagents).
(e) Degassing time (the degassing time with nitrogen was reduced by half).

In Table 2 are shown the values used in each experiment. It is important to notice that experiment 7 corresponds to the predetermined conditions with similar results to those already presented in literature, thus no results are presented here.

#### 2.2.4. Economic Analysis and Industrial Scale-Up Study

The following methodological steps were applied for the economic analysis and industrial scale-up [8]: (a) determine the optimized process for magnetic hyperthermia; (b) perform a market study to determine the demand of the product (magnetic nanoparticles) for hyperthermia applications; (c) determine the size of the plant; (d) analyze all the process steps, choose all the process units and perform all the mass and energy balances required in the plant; (e) detailed scale-up design of the main equipment; and (f) analyze the global economic impact and profitability.

### 2.3. Instrumentation 

The main instrumentation used was: Three-way Reactor (Nahita, Madrid), Orbital stirrer (Nahita, Madrid), Thermal Heater (Nahita, Madrid), Centrifuge (Fischer, Barcelona), besides regular laboratory glass material.

## 3. Results and Discussion

In order to determine the best set of values for the main producing parameters, the obtained particles need to be analyzed and compared according to their main characteristics. In this context, it is important to analyze: magnetic properties (magnetic susceptibility), phase composition or phase distribution ratio (XRD), particle size, and morphology (SEM).

### 3.1. Magnetic Properties

In Table 3 are shown the results obtained for the magnetic properties of the final product corresponding to each experiment.

As it may be seen, the large majority of the obtained nanoparticles present the same order of magnitude for the magnetic susceptibly. However, particles obtained in experiments 2, 11, 12, and 14 present the highest values, while the lowest values were obtained for experiments 7 and 10.

### 3.2. Composition

**Figure 2:**
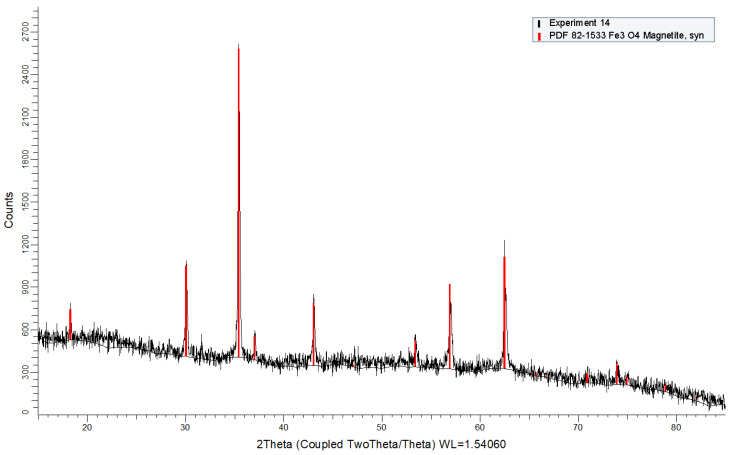
X-ray of experiment 14.

Concerning their structure, X-ray analysis were made on all particles obtained from the set of the performed experiments. In Figure 2 is presented one of the diffraction patterns obtained for these. They all are almost coincident and present the same peaks, so they may be represented by the one depicted in Figure 2 (except for experiment 15 and 16 for which no analysis was possible to be done due to the nature of the obtained products). The typical magnetite peaks are shown in red, according to Powder Diffraction File PDF2 dataset #82-1533 (Powder Diffraction File, International Centre for Diffraction Data, Newtown Square, PA, USA). No additional peaks are found, so samples are essentially pure magnetite.

In addition to the qualitative analysis, an estimation of the crystallite size was calculated by using the Scherrer equation (Table 4). The main peak (at ca. 35.5°) was used for the calculations. Lanthanum hexaboride (NIST-660b) was used as crystalline standard for instrumental broadening.

### 3.3. Particle Size and Morphology

**Figure 3:**
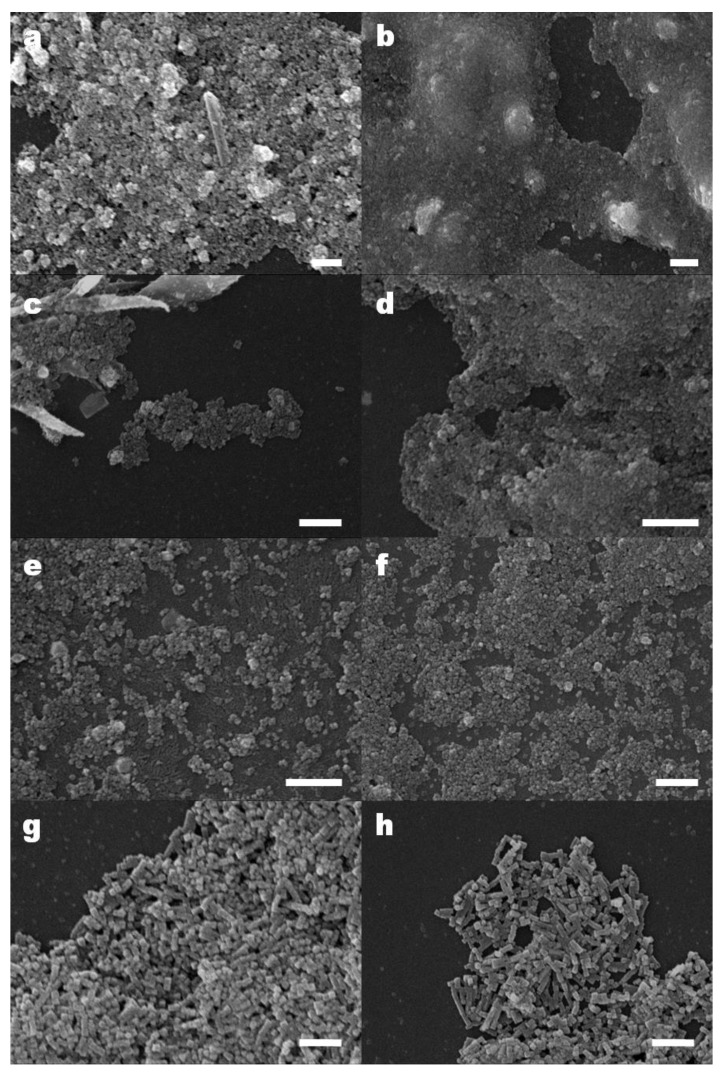

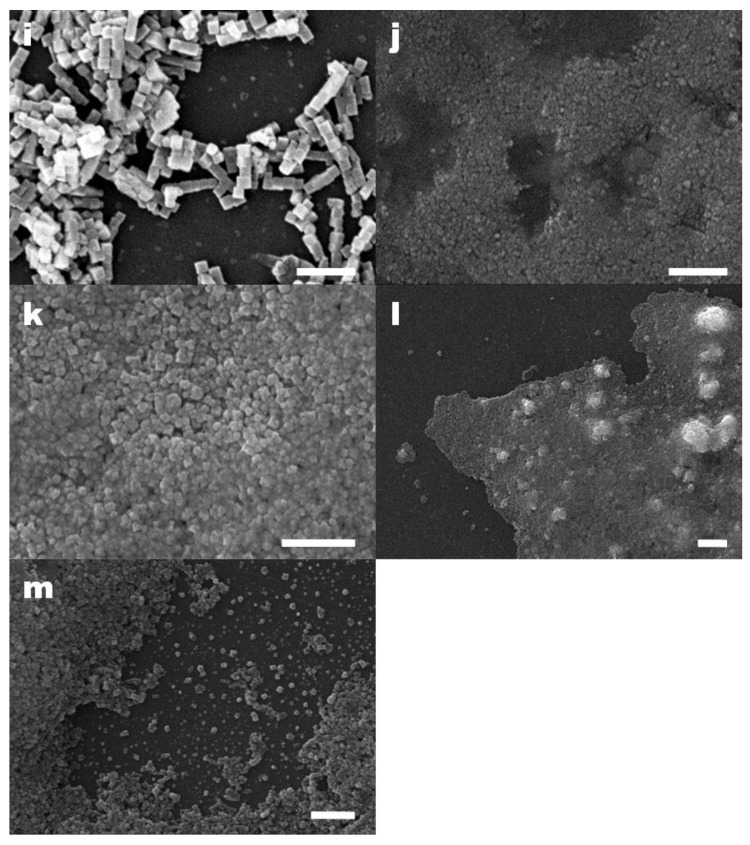
SEM of the nanomagnetite particles obtained in experiments: (**a**) 1; (**b**) 2; (**c**) 3; (**d**) 4; (**e**) 5; (**f**) 6; (**g**) 7; (**h**) 8; (**i**) 9; (**j**) 10; (**k**) 11; (**l**) 13; (**m**) 12 and 14. White bar length = 1 µm.

In Figure 3 are presented the secondary electron SEM images of the particles obtained in experiments 1–14, while in Table 5 are presented their size ranges percentages, extracted from the corresponding histograms shown in Supplementary Materials. For some particles, high-quality images were not possible to obtain, mostly due to charging, even after gold sputter coating. In spite of this setback, relevant data were obtained, as summarized in Table 5 and Table 6. Some general comments may be emphasized. Typically, when poorly shaped particles are obtained, some tendency to large agglomeration is present, maybe suggesting some kind of growth interference (e.g., Figure 3a,d). When moderately shaped particles are attained, there is a tendency for euhedral forms, almost cubic, though occasional octahedra may be present (e.g., some triangles in Figure 3k), but no rhombic-dodecahedra have been detected. At last, when well-shaped forms are obtained, there is an undoubted preference for cubes and parallelepipeds, in accordance to the results of Kim et al. 2008 [24]. Some stacking is observed (e.g., Figure 3g–i), but it has to be underlined that twinning is not detected in any well-shaped sample.

### 3.4. Discussion of Results

In Table 6 is presented a summary of the main conclusions, considering all the results presented previously.

From Table 3, it may be concluded that except for the case of experiments 15 and 16 (where no particles are obtained), the particles present the same order of magnitude concerning magnetic properties (being experiments 2, 11, 12, and 14 the ones that present particles with higher magnetic properties and 7 and 10 the ones that present particles with lower magnetic properties, by a ratio of 3–4). Clearly, the process is well behaved for the majority of parameters, does not pose serious threat to the attainment of magnetite crystals, and does not have a strong influence on their anisotropy and the alignment of magnetic moments. The exceptions are experiments 15 and 16, corresponding to the increase of the surfactant (oleic acid) and one of the tested solvents (4-biphenylcarboxylic acid), respectively. 

Considering the attainment of the cubic shape, only experiments 3 (highest stirring), 6 (highest ramp-up temperature scale gradient), 10 (highest centrifuging speed), and 11 (doubling of all reagents quantities) are acceptable, and experiments 7, 8, 9 (all using benzyl ether as solvent), 12, and 14 (using both solvents—benzyl ether and 4-biphenylcarboxylic acid, highest centrifuging speed and high temperature ramp-up gradient) are ideal. Comparing the products of these last experiments, considering the size of the particles, it may be concluded that particles obtained in experiments 7, 8, and 9 present a much larger size than desired (using benzyl ether as solvent alone is good for the shape, but not for obtaining small sizes); in experiments 3, 6, 10, and 11 the obtained particles present slightly larger size than desired and present agglomerations, while in experiments 12 and 14, obtained particles present the desired size and no agglomerations are observed. Therefore, steps followed to obtain particles in experiments 12 and 14 stand-out as the optimized particle production method, as these experiments present final cubic-shaped nanoparticles with high magnetic properties, with the desired size and no agglomerations. Hence, using both solvents at the same time (benzyl ether and 4-biphenylcarboxylic acid) and maintaining the selected base preferences for the other reagents and stirring speed (while increasing the centrifuging speed) seems to be the best option to obtain the desired magnetic nanoparticles. 

#### 3.4.1. Parameters Influence

By analyzing each parameter, it may be concluded that:

(a) increasing stirring speed is beneficial (experiments 1, 2, and 3),
(b) increase in temperature ramp-up does not affect the results (experiments 4, 5, 6, 7, 8, 9, and 10),
(c) substituting the solvent (4-biphenylcarboxylic acid by benzyl ether) improves the percentage of particles presenting a cubic form, but increases particle sizes (experiments 7, 8, and 9),
(d) doubling the quantities of oleic acid or 4-biphenylcarboxylic acid leads to a null production of particles (experiments 15 and 16),
(e) doubling the quantity of the two reactives and one of the tested solvents is not detrimental (experiment 11),
(f) reducing the degasification time is detrimental (experiment 13),
(g) using both solvents at the same time (4-biphenylcarboxylic acid and benzyl ether) improves the process (experiments 12 and 14).

#### 3.4.2. Ideal Value of the Parameters

From all the above, it may be concluded that the ideal conditions are: iron acetylacetonate (III)—2 mmol; oleic acid—4.5 mmol; 4-biphenylcarboxylic acid—2 mmol; benzyl ether—52.5 mmol; stirring—200 rpm; reaction temperature—290 °C; temperature ramp-up—25 °C/min; centrifugal speed 2500 rpm.

## 4. Potential Industrial Implementation

To determine the potential industrial implementation of the cubic magnetic nanoparticle manufacturing process, the methodology described in [8] is followed: first, determine the potential international demand of the product (magnetic nanoparticles for magnetic hyperthermia applications), then, determine the plant size, analyze all the process steps and chose all the process units (based on the mass and energy balances), perform a detailed scale-up design of the main equipment, and finally analyze the economic impact and profitability. By following this methodology it was possible to determine the technological and economic viability of the industrial implementation of the optimized process for production of cubic-shaped magnetite nanoparticles based on the optimized values found in Section 3. In what follows, only the main results are present (in Supplementary Material are given all the details concerning these calculations).

### 4.1. Size of the Plant

The minimum size of the plant in order to be profitable is 3850 kg/year.

The plant will be designed for an annual income of EUR 203,200,000, at a price of EUR 30,000/kg magnetic nanoparticles. The annual production is equivalent to 6773 kg/year.

### 4.2. Process Engineering Design

**Figure 4:**
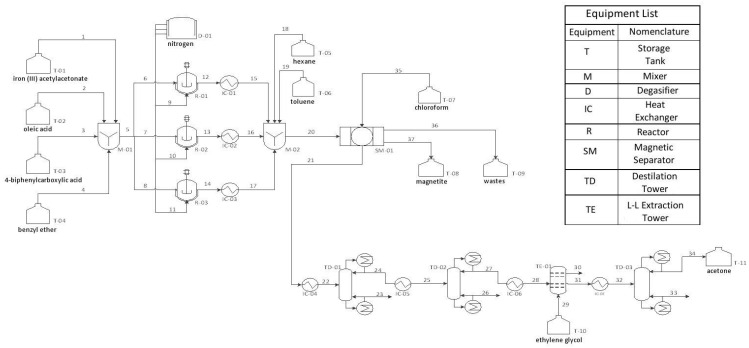
Process Engineering Flowsheet of the Plant to Produce Cubic-Shaped Magnetite Nanoparticles.

In Figure 4 is depicted the process diagram of the proposed industrial plant and in Supplementary Material the characteristics of each stream.

The process begins in the M-01 mixer where the main components (stored in the T-01, T-02, T-03, and T-04 tanks) are mixed in their stoichiometric proportions. The main raw materials are: iron (III) acetylacetonate, which will act as the precursor agent, oleic acid, which will be the surfactant and intervenes in the ligand exchange reaction with iron acetylacetonate, and 4-biphenylcarboxylic acid and benzyl ether, which will act as solvents. When the raw materials have been mixed in the M-01, the mixture is taken to the R-01 reactor, as well as to reactors R-02 and R-03. In the reactors, the degassing is done with nitrogen delivered by the degasser D-01. The degassed mixture is then heated through a jacket heater up to 290 °C (563 K), with a constant temperature ramp-up rate of 25 °C/min and a stirring of 800 rpm. When the mixture reaches 290 °C, it remains constant for 30 min. Then, the sample is fed to the heat exchanger IC01, where it is cooled to room temperature. Subsequently, the mixture is introduced in the mixing container M-02 and a mixture of hexane and toluene is added, from tanks T-05 and T-06, with a ratio of 1:4, to facilitate the subsequent separation. The entire mixture is then passed through a magnetic separator SM-01, which is responsible for retaining the MNPs synthesized in the reactor. Lastly, the MNPs are cleaned with chloroform and stored in tank T-07. Regarding the remaining compounds, they are discarded by the magnetic separator through stream 21, including benzyl ether, hexane, toluene, and the other by-products (acetone), which are recycled to reduce raw material costs. For such an effect, distillation towers and a liquid-liquid extraction tower are used.

### 4.3. Global Economic Impact and Profitability

In Table 7 is presented a summary of the overall economic balance of the plant.

As it may be seen from Table 7 and the calculations in the Supplementary Material, a maximum net benefit of EUR 100,462,182/year and a margin of profits over costs of 145% may be attained.

### 4.4. Discussion of Results—Industrial Adaptation

From the obtained results, the economic viability of the industrial implementation of a plant producing magnetic nanoparticles for hyperthermia applications seems evident. Nonetheless, it is important to notice that market demand was based on the current and foreseen demand for biomedical applications. This means that the produced particles must also be able to be used in other biomedical applications in order to reach the expected selling amount and price. The characteristics of the particles seem appropriate for this goal also (although in some cases it will probably require a further process stage for functionalization, but the economic margin is enough to absorb this step and maintain economic viability). 

In what concerns the technological viability, the designed process seems appropriate, also considering all the mass and heat balances that were performed and the process units applied. The recovery and recycling of the raw materials after reaction is considered, as well as an appropriate recovery and purification step concerning the produced magnetic nanoparticles.

## 5. Conclusions

Hyperthermia applications of magnetic nanoparticles is currently a highly intensive field of research. Cubic-shaped magnetite particles seem to be the most promising type of particles for magnetic hyperthermia. Nonetheless, only a few studies present proper ways to obtain this type of particle, no optimization of the processes has been done, and no proper study of the influence of the main operating parameters is available in the current literature. In this work, a method for the production of cubic-shaped magnetite nanoparticles was applied and the influence of the solvent, precursor and surfactant quantities and ratio, type of solvent, stirring speed, centrifuge speed, temperature ramp-up gradient, reaction temperature, and degassing time was studied. It was concluded that increasing stirring speed is beneficial, an increase in temperature ramp-up does not affect the results, substituting the solvent (4-biphenylcarboxylic acid by benzyl ether) improves cubic form presence but increases particle size, doubling the quantities of oleic acid or 4-biphenylcarboxylic acid leads to a null production of particles, doubling the quantity of the two reactives and one of the solvents is not detrimental, reducing the degasification time is detrimental, and using both solvents at the same time (4-biphenylcarboxylic acid and benzyl ether) improves the process. The best values for the studied variables are: iron acetylacetonate (III)—2 mmol; oleic acid—4.5 mmol; 4-biphenylcarboxylic acid—2 mmol; benzyl ether—52.5 mmol; stirring—200 rpm; reaction temperature—290 °C; temperature ramp-up—25 °C/min; centrifugal speed—2500 rpm.

Then, the industrial implementation of a process to produce these magnetite nanoparticles based on the optimized values reached in the research stage was fully designed and studied in order to study the potential industrial implementation viability. Full process engineering, including energy and mass balances, was also conducted for the plant. The designed plant has proven to be viable economically and technically, with a maximum net benefit of EUR 100,462,182/year. Since the profit is much larger than regular bank benefits, investors may consider attractive the construction and startup of the designed industrial plant, even if several of the parameters have to be adjusted (e.g., adjustment of the sell price of the particles, estimation of some costs, etc.). It is expected that this work will serve as a demonstration of the connection between science and practical applications, and to allow scientific improvement of other processes bearing in mind their possible industrial implementation.

## Figures and Tables

**Table 1:** Comparison between selected magnetic nanoparticle manufacturing methods.

Manufacturing Method	Synthesis	Reaction Temp. (°C)	Reaction Time	Solvent	Surface-Capping Agents	Size Distribution	Shape Control	Yield
Coprecipitation	Very simple	20–90	Minutes	Water	During/after reaction	Relatively narrow	Not good	High
Microemulsion	Complicated	20–50	Hours	Organic agents	During reaction	Relatively narrow	Good	Low
Thermal decomposition	Complicated	100–320	Hours-days	Organic agents	During reaction	Very narrow	Very good	High
Hydrothermal	Simple	200–250	Hours-days	Water-ethanol	During reaction	Very narrow	Very good	Medium
Poliol	Simple	25-Boiling Point	Hours	Ethylene PEG	During reaction	Narrow	Very Good	High

**Table 2:** Experiments done for optimization of the synthesis process (when the value of a specific parameter differs from the default, the value of the parameter is highlighted in grey).

	Reagent/Property								
Experiment	Iron Acetylacetonate (III) (mmol)	Oleic Acid (mmol)	4-Biphenylcarboxylic Acid (mmol)	Benzyl ether (mmol)	Reaction Temperature (°C)	Temperature Ramp-Up (°C/min)	Stirring Speed (rpm)	Centrifuge (rpm)	Degassing
1	2	4.5	2	-----	290	20	150	1700	Normal
2	2	4.5	2	-----	290	20	170	1700	Normal
3	2	4.5	2	-----	290	20	220	1700	Normal
4	2	4.5	2	-----	290	25	220	2000	Normal
5	2	4.5	2	-----	290	15	220	2000	Normal
6	2	4.5	2	-----	290	30	220	2000	Normal
7	2	4.5	-----	52.5	290	15	200	2500	Normal
8	2	4.5	-----	52.5	290	20	200	2500	Normal
9	2	4.5	-----	52.5	290	25	200	2500	Normal
10	2	4.5	2	-----	290	20	220	2500	Normal
11 (*)	4	9	4	-----	290	25	200	2500	Normal
12 (**)	2	4.5	2	52.5	290	25	200	2500	Normal
13	2	4.5	2	-----	290	30	200	2500	Half
14	2	4.5	2	52.5	290	25	200	2500	Normal
15	2	9	2	-----	290	20	200	2500	Normal
16	2	4.5	4	-----	290	30	200	2500	Normal

(*): It is important to notice that experiment 11 is not an upscale version of experiment 14. In fact, the only parameters that were doubled in experiment 11 were the reagents and one of the solvents, and not all the remaining parameters (to perform an upscale all the other parameters should be also modified accordingly—as done in the upscale and economic analysis presented at the final section of the paper). (**) Preliminary study of experiment 14 with coincident results.

**Table 3:** Magnetic properties of the produced nanoparticles.

Experiment Number	1	2	3	4	5	6	7	8
Magnetic susceptibility (SI)	2.51 × 10^−4^	5.29 × 10^−4^	2.84 × 10^−4^	1.56 × 10^−4^	1.51 × 10^−4^	2.56 × 10^−4^	9.71 × 10^−5^	2.00 × 10^−4^
Experiment number	9	10	11	12 and 14	13	15	16	
Magnetic susceptibility (SI)	2.13 × 10^−4^	9.25 × 10^−5^	4.64 × 10^−4^	3.12 × 10^−4^	2.44 × 10^−4^	-----	-----	

**Table 4:** Estimation of the crystallite size (calculated by using the Scherrer equation).

Experiment Number	1	2	3	4	5	6	7	8
D (nm)	73	50	44	39	32	37	88	92
**Experiment number**	**9**	**10**	**11**	**12 and 14**	**13**	**15**	**16**	
D (nm)	121	36	54	32	50	-----	-----	

**Table 5:** Size ranges percentages of each experiment product. The size ranges showing highest percentage are highlighted for each case: light grey—between 10–19%; medium gray-between 20–29%; dark grey—more than 30%.

Exp. Number	10–20 (nm)	20–28 (nm)	28–36 (nm)	36–44 (nm)	44–52 (nm)	52–60 (nm)	60–68 (nm)	68–76 (nm)	76–84 (nm)	84–92 (nm)	92–100 (nm)	>100 (nm)
**1**	0%	0%	0%	0%	0%	0%	1%	2%	3%	2%	12%	80%
**2**	0%	0%	0%	0%	2%	8%	8%	9%	14%	14%	13%	32%
**3**	0%	0%	4%	15%	24%	27%	15%	12%	1%	0%	2%	0%
**4**	0%	5%	12%	28%	26%	18%	7%	2%	0%	2%	0%	0%
**5**	0%	3%	12%	25%	26%	14%	9%	6%	3%	1%	2%	0%
**6**	0%	6%	24%	25%	17%	17%	5%	2%	2%	2%	0%	0%
**7**	0%	0%	0%	0%	1%	4%	4%	11%	12%	12%	10%	46%
**8**	0%	0%	0%	0%	0%	1%	1%	3%	3%	3%	7%	82%
**9**	0%	0%	0%	0%	0%	0%	0%	0%	1%	2%	4%	93%
**10**	0%	11%	9%	19%	30%	18%	9%	4%	0%	0%	0%	0%
**11**	0%	4%	13%	18%	16%	20%	10%	13%	2%	3%	2%	0%
**12, 14**	6%	26%	32%	16%	9%	5%	4%	1%	1%	0%	0%	0%
**13**	0%	4%	6%	6%	15%	20%	24%	12%	5%	5%	4%	3%
**15**	----	----	----	----	----	----	----	----	----	----		
**16**	----	----	----	----	----	----	----	----	----	----		

**Table 6:** Main conclusions from the obtained results.

Exp. Number	Morphology	Main Size Ranges	Magnetic Character	Conclusions
1	Undefined	92–100 nm: 12% >100 nm: 80%	Regular	The low resolution does not allow to determine their precise morphology. Size much larger than desired. Presents some large agglomerations.
2	Undefined	76–84 nm: 14% 84–92 nm: 14% 92–100 nm: 13% >100 nm: 32%	Slightly Higher	The low resolution does not allow to determine their precise morphology. Size much larger than desired. Presents some large agglomerations.
3	Generally irregular. Some cubic-shaped particles are present.	36–44 nm: 15% 44–52 nm: 24% 52–60 nm: 27% 60–68 nm: 15% 68–76 nm: 12%	Regular	Acceptable morphology. Size larger than desired. Some large agglomerations are present.
4	Irregular	28–36 nm: 12% 36–44 nm: 28% 44–52 nm: 26% 52–60 nm: 18%	Regular	Poorly defined morphology. Size slightly larger than desired. Small agglomerations of medium size are seen.
5	Irregular	28–36 nm: 12% 36–44 nm: 25% 44–52 nm: 26% 52–60 nm: 14%	Regular	Poorly defined morphology. Size slightly larger than desired. Small agglomerations of medium size are seen.
6	Generally irregular. Some cubic-shaped particles are present.	28–36 nm: 24% 36–44 nm: 25% 44–52 nm: 17% 52–60 nm: 17%	Regular	Acceptable morphology. Size slightly larger than desired. Some agglomerations of medium size are seen.
7	Cubic/cobblestone particles	68–76 nm: 11% 76–84 nm: 12% 84–92 nm: 12% 92–100 nm: 10% >100 nm: 46%	Slightly Lower	Ideal morphology. Size much larger than desired. There are no agglomerations.
8	Cubic/cobblestone particles	>100 nm: 82%	Regular	Ideal morphology. Size much larger than desired. There are no agglomerations.
9	Cubic/cobblestone particles	>100 nm: 93%	Regular	Ideal morphology. Size much larger than desired. There are no agglomerations.
10	Generally irregular. Some cubic-shaped particles are present.	20–28 nm: 11% 28–36 nm: 9% 36–44 nm: 19% 44–52 nm: 30% 52–60 nm: 18%	Slightly Lower	Acceptable morphology. Size slightly larger than desired. Large agglomerations are not visible.
11	Generally irregular. Some cubic-shaped particles are present.	28–36 nm: 13% 36–44 nm: 18% 44–52 nm: 16% 52–60 nm: 20% 60–68 nm: 10% 68–76 nm: 13%	Slightly Higher	Acceptable morphology. Size slightly larger than desired. There are several medium-sized agglomerations
13	Undefined	44–52 nm: 15% 52–60 nm: 20% 60–68 nm: 24% 68–76 nm: 12%	Regular	The low resolution does not allow to determine their precise morphology. Size much larger than required. Presents some large agglomerations.
12 and 14	Generally cubic-shaped particles are present. Some irregular shape also appears.	20–28 nm: 26% 28–36 nm: 32% 36–44 nm: 16%	Slightly Higher	Very good morphology. Very good size. There are no agglomerations.
15	No particles obtained	-----------	-----------	-----------
16	No particles obtained	-----------	-----------	-----------

**Table 7:** Overall economic balance of industrial implementation of MNPs production for magnetic hyperthermia applications.

Cost	EUR	Invested Capital	EUR
1.1 Raw Materials	48,298,030	1.1 Instrumentation	827,043
1.2 Direct Human Labor	829,870	1.2 Initial Setup	473,602
1.3 Indirect Human Labor	207,358	1.3 Piping and Valves	372,270
1.4 General Services	580,909	1.4 Measuring and Control	165,409
1.5 Supplies	80,096	1.5 Heat Isolation	57,893
1.6 Maintenance	234,179	1.6 Electrical Installation	124,057
1.7 Laboratory	165,974	1.7 Land and Structures	1,122,893
1.8 Board and Technical Staff	224,564	1.8 Auxiliary Facilities	330,817
1.9 Amortization	82,704	1.9 Project and Design	361,923
1.10 Taxes and Insurances	160,192	1.10 Constructor Hiring	208,439
TOTAL COST OF FABRICATION	50,863,876	1.11 Unexpected Expenses	521,098
2.1 Commercial Expenses	10,172,775	1.12 Preliminary Studies	2,803,343
2.2 Management	131,826	1.13 Preliminary Startup	640,764
2.3 Financial Expenses	5,809,661	-	-
2.4 Research	240,286	TOTAL IMMOBILIZED	8,009,550
2.5 Technical Services	2,032,000	CIRCULATING CAPITAL	21,038,753
TOTAL COST OF MANAGEMENT	18,386,548	TOTAL INVESTED CAPITAL	29,048,303
TOTAL PRODUCTION COSTS	69,250,424	TOTAL INCOME	203,200,000

## Data Availability

Not applicable.

## Supplementary Materials

The following are available online at https://www.mdpi.com/article/10.3390/nano11071652/s1, Figure S1: Histogram and size range of the synthesized nanomagnetic particles—Sample 1.; Figure S2: Histogram and size range of the synthesized nanomagnetic particles—Sample 2.; Figure S3: Histogram and size range of the synthesized nanomagnetic particles—Sample 3.; Figure S4: Histogram and size range of the synthesized nanomagnetic particles—Sample 4.; Figure S5: Histogram and size range of the synthesized nanomagnetic particles—Sample 5.; Figure S6: Histogram and size range of the synthesized nanomagnetic particles—Sample 6.; Figure S7: Histogram and size range of the synthesized nanomagnetic particles—Sample 7.; Figure S8: Histogram and size range of the synthesized nanomagnetic particles—Sample 8.; Figure S9: Histogram and size range of the synthesized nanomagnetic particles—Sample 9.; Figure S10: Histogram and size range of the synthesized nanomagnetic particles—Sample 10.; Figure S11: Histogram and size range of the synthesized nanomagnetic particles—Sample 11.; Figure S12: Histogram and size range of the synthesized nanomagnetic particles—Sample 13.; Figure S13: Histogram and size range of the synthesized nanomagnetic particles—Sample 12 and 14.; Figure S14: Market growth for nanoparticles for biomedical applications.; Figure S15: Nanomedicine market growth rate until 2019.; Figure S16: Another study of the nanomedicine market until 2020.; Figure S17: Total costs/incomes *versus* total capacity; Table S1: Costs of Raw Materials; Table S2: Direct human labor costs.; Table S3: Labor costs for indirect human labor.; Table S4: Chief Personnel labor costs; Table S5: Management Personnel labor costs; Table S6: Streams of the Plant and their characteristics.
