# Supplementary material for: Cubic Nanoparticles for Magnetic Hyperthermia: Process Optimization and Potential Industrial Implementation
Source: Nanomaterials (Basel). 2021 Jun 23;11(7):1652. doi: 10.3390/nano11071652 (PMC8306292; doi:10.3390/nano11071652)
Supplement: Supplementary file 1 [file nanomaterials-11-01652-s001.zip › nanomaterials-1111059-supplementary.pdf]

nanomaterials

MDPI

Article

Cubic Nanoparticles for Magnetic Hyperthermia: Process Optimization and Potential Industrial Implementation

Omar Sánchez Sánchez [1], Teresa Castelo-Grande [2], Paulo A. Augusto [1,3,*], José M. Compaña [4] and Domingos Barbosa [2]

[1] Departamento de Ingeniería Química y Textil, Facultad de Ciencias Químicas, Universidad de Salamanca, Plaza de los Caídos, 1-5, 37008 Salamanca, Spain; pauloaugusto@usal.es
[2] LEPABE—Laboratory for Process Engineering, Environment, Biotechnology and Energy, Faculty of Engineering, University of Porto, Rua Dr. Roberto Frias, 4200-465 Porto, Portugal; tcg@fe.up.pt (T.C.-G.); dbarbosa@fe.up.pt (D.B.)
[3] Instituto de Biología Molecular y Celular del Cáncer, CSIC/Universidad de Salamanca (GIR Citómica), Campus Miguel de Unamuno, 37007 Salamanca, Spain
[4] Servicio de Difracción de Rayos-X, Universidad de Salamanca, Pza. de Los Caídos s/n, 37008 Salamanca, Spain; jmcompana@usal.es
* Correspondence: pauloaugusto@usal.es

Abstract: Cubic nanoparticles are referred to as the best shaped particles for magnetic hyperthermia applications. In this work, the best set of values for obtaining optimized shape and size of magnetic particles (namely: reagents quantities and proportions, type of solvents, temperature, etc.) is determined. A full industrial implementation study is also performed, including production system design and technical and economic viability.

Keywords: magnetic hyperthermia; magnetic nanoparticles; optimization; economic analysis; plant design and process engineering; cubic particles

Several processes exist to produce magnetic nanoparticles. In the case of hyperthermia applications, cubic shape particles are currently the most advisable. Some methods have been developed to manufacture these type of particles. However, no study considering the influence of the most important process parameters, and thus no optimization of the manufacturing process has been presented yet. In this Supplementary Material complementary information is given concerning the research performed, bearing in mind the optimization goal. Besides, details of the results of the economic viablitity study presented in the main text are also given.

Considering the economic study, it is important to notice that precise quantificaction of the expenses related to any plant, when designing them, is impossible to know. Hence, estimations are always the best solutions, especially at the design or upscale stage. Several scientific accepted methods exist in literature to guide the ways to achieve a proper estimation, which have been followed (e.g. [1–3]). Nonetheless, these approaches must be adapted considering the type of plant and product in question as different requisites are imposed. Hence, throughout all this work, in the calculus of each expense or profit it has been applied the best estimation always considering as goal the production of cubic sized magnetic nanoparticles for hyperthermia applications.

A – Experimental Procedure

In Figure S1 are depicted photographs of the steps taken to carry out the experiment.

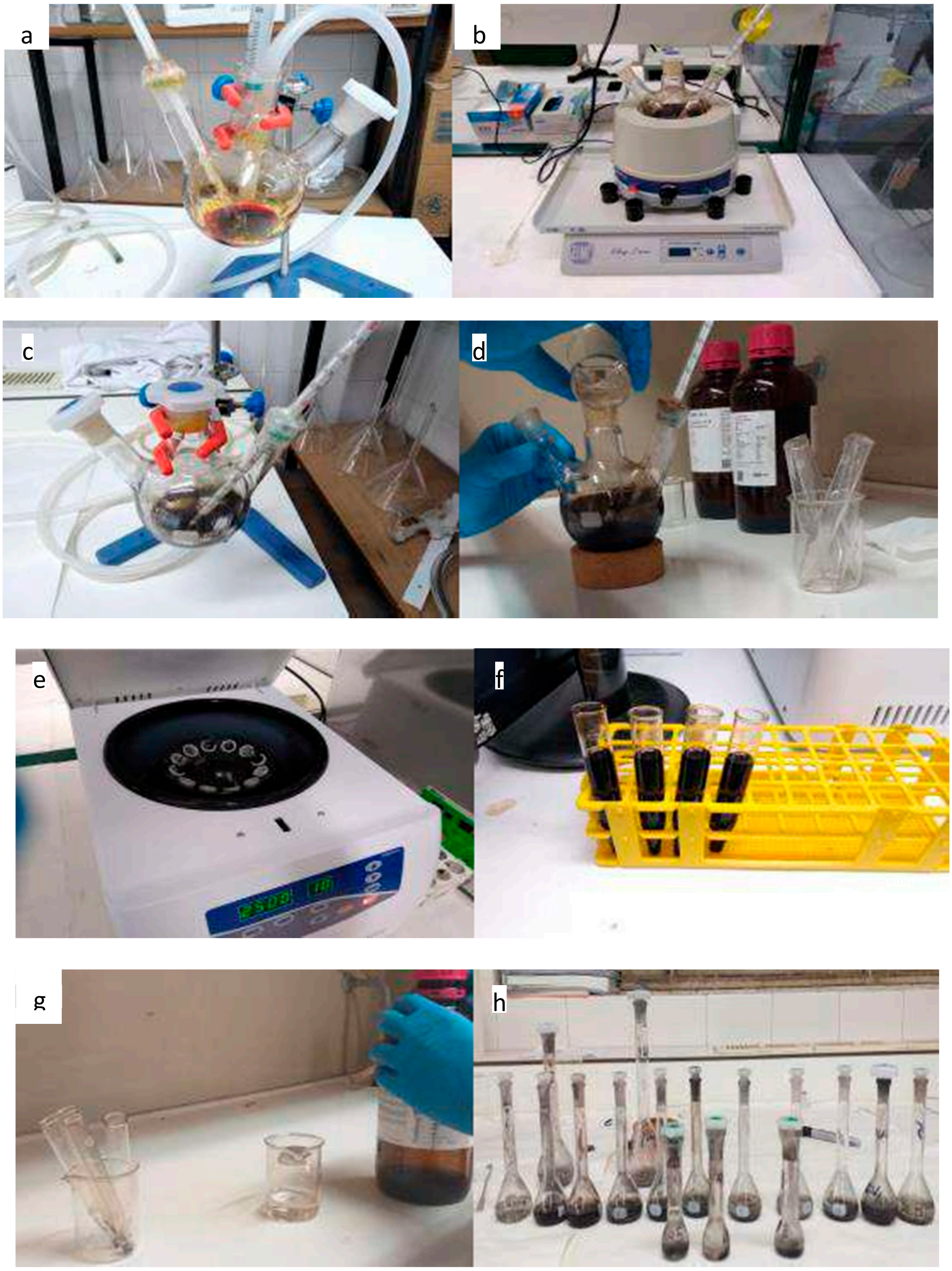

Figure S1. - Different stages of the process: a) Degassing, b) Heating, c) Cooling, d) Adding Toluene and Hexane, e) Centrifuging, f) Centrifuged samples, g) Eliminating waste and cleaning with chloroform, h) Synthesized nanoparticles in chloroform.

In Figure S2 are shown some photographs of the samples of the synthesized nano-magnetic particles

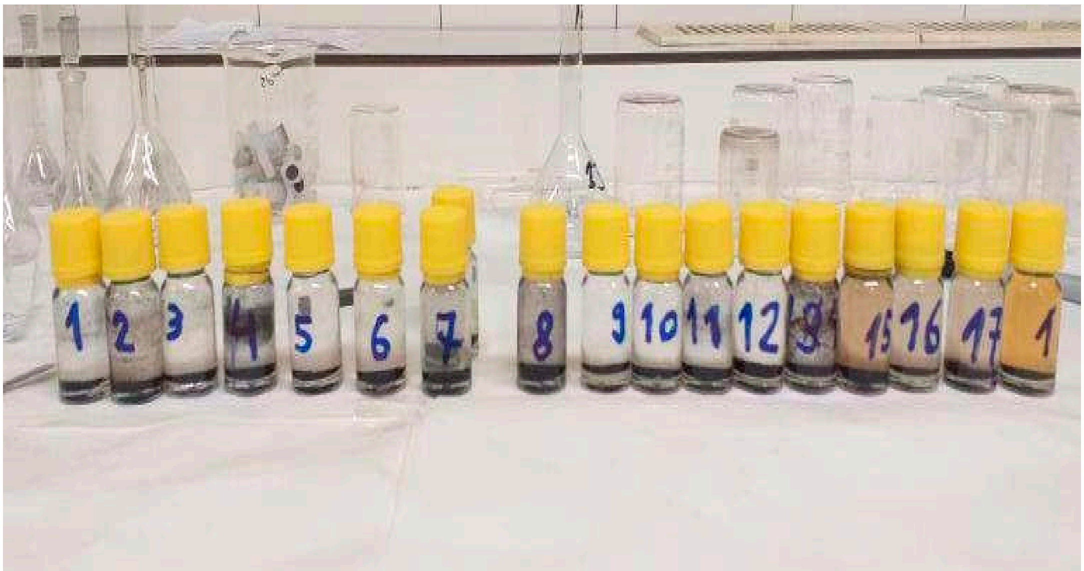

Figure S2. - Collection of some samples in chloroform (the numbers do not correspond to the experiment numbers presented in this article).

B – Determination of the influence of the main parameters

In Figures S3 to S15 are shown the results obtained concerning the size of the particles.

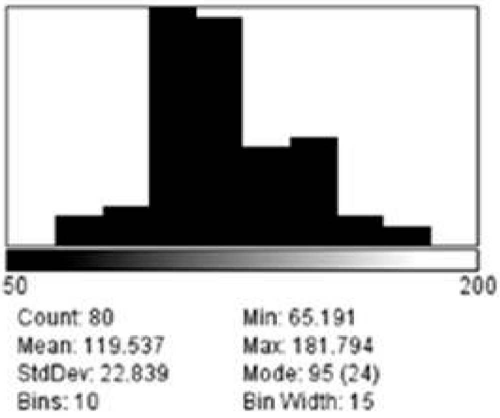

Size range of particles (nm)	Frequency	%
50-65	0	0
65-80	3	4
80-95	4	5
95-110	24	30
110-125	23	29
125-140	10	13
140-155	11	14
155-170	3	4
170-185	2	3
185-200	0	0
TOTAL	80	100

Figure S3. – Histogram and size range of the synthesized magnetic nanoparticles – Experiment 1.

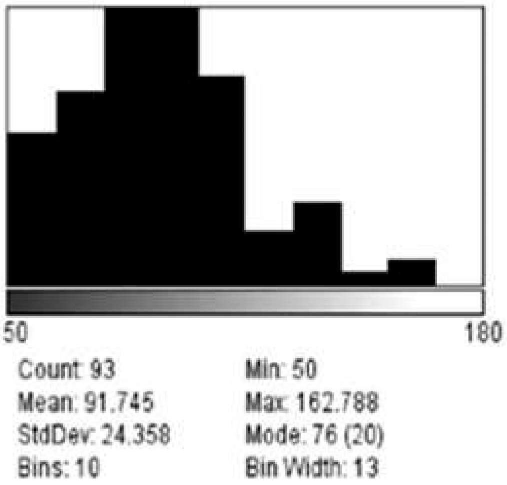

Size range of particles (nm)	Frequency	%
50-63	11	12
63-76	14	15
76-89	20	22
89-102	20	22
102-115	15	16
115-128	4	4
128-141	6	6
141-154	1	1
154-167	2	2
167-180	0	0
TOTAL	93	100

Figure S4. – Histogram and size range of the synthesized magnetic nanoparticles – Experiment 2.

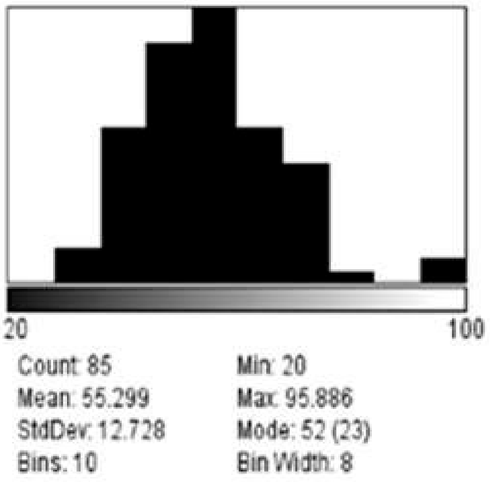

Size range of particles (nm)	Frequency	%
20-28	0	0
28-36	3	4
36-44	13	15
44-52	20	24
52-60	23	27
60-68	13	15
68-76	10	12
76-84	1	1
84-92	0	0
92-100	2	2
TOTAL	85	100

Figure S5. – Histogram and size range of the synthesized magnetic nanoparticles – Experiment 3.

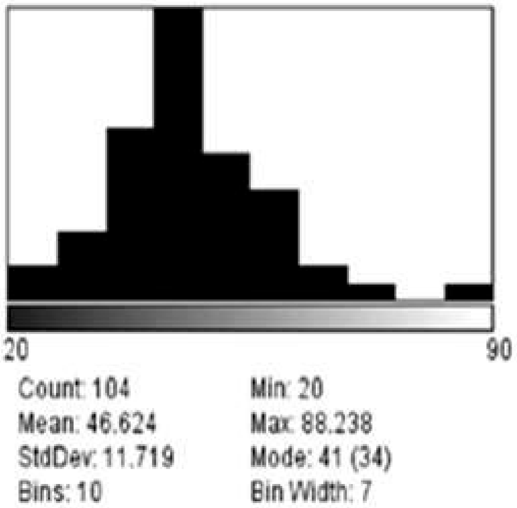

Size range of particles (nm)	Frequency	%
20-27	4	4
27-34	8	8
34-41	20	19
41-48	34	33
48-55	17	16
55-62	13	13
62-69	4	4
69-76	2	2
76-83	0	0
83-90	2	2
TOTAL	104	100

Figure S6. – Histogram and size range of the synthesized magnetic nanoparticles – Experiment 4.

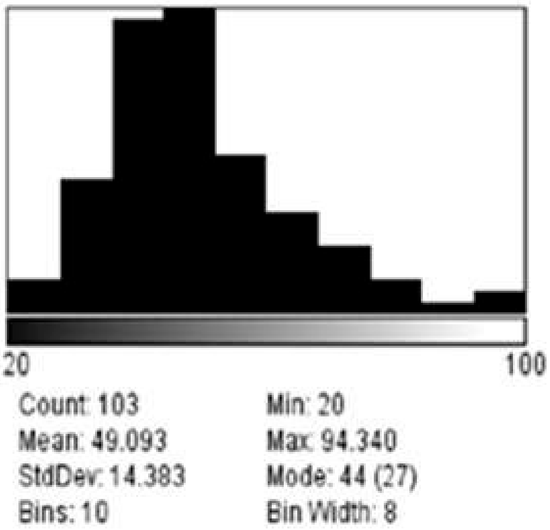

Size range of particles (nm)	Frequency	%
20-28	3	3
28-36	12	12
36-44	26	25
44-52	27	26
52-60	14	14
60-68	9	9
68-76	6	6
76-84	3	3
84-92	1	1
92-100	2	2
TOTAL	103	100

Figure S7. – Histogram and size range of the synthesized magnetic nanoparticles – Experiment 5.

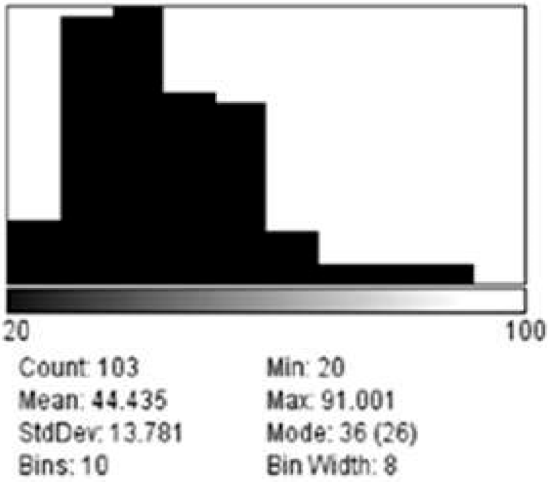

Size range of particles (nm)	Frequency	%
20-28	6	6
28-36	25	24
36-44	26	25
44-52	18	17
52-60	17	17
60-68	5	5
68-76	2	2
76-84	2	2
84-92	2	2
92-100	0	0
TOTAL	103	100

Figure S8. – Histogram and size range of the synthesized magnetic nanoparticles – Experiment 6.

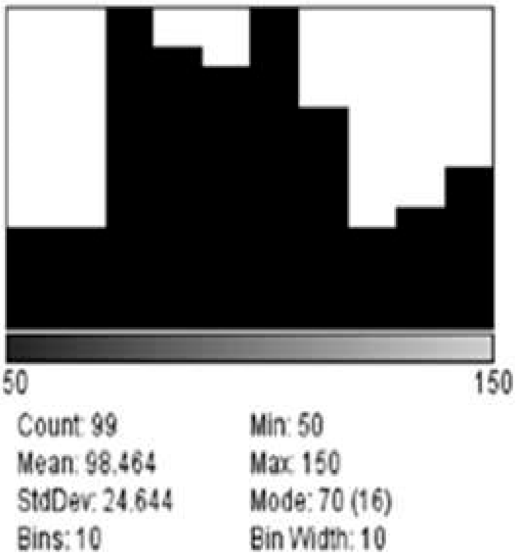

Size range of particles (nm)	Frequency	%
50-60	5	5
60-70	5	5
70-80	16	16
80-90	14	14
90-100	13	13
100-110	16	16
110-120	11	11
120-130	5	5
130-140	6	6
140-150	8	8
TOTAL	99	100

Figure S9. – Histogram and size range of the synthesized magnetic nanoparticles – Experiment 7.

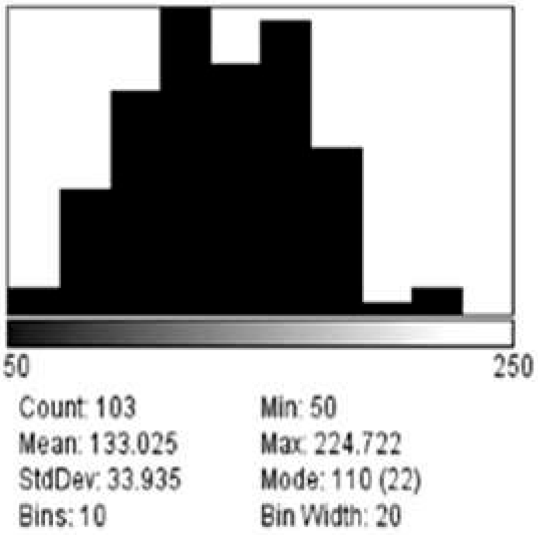

Size range of particles (nm)	Frequency	%
50-70	2	2
70-90	9	9
90-110	16	16
110-130	22	21
130-150	18	17
150-170	21	20
170-190	12	12
190-210	1	1
210-230	2	2
230-250	0	0
TOTAL	103	100

Figure S10. – Histogram and size range of the synthesized magnetic nanoparticles – Experiment 8.

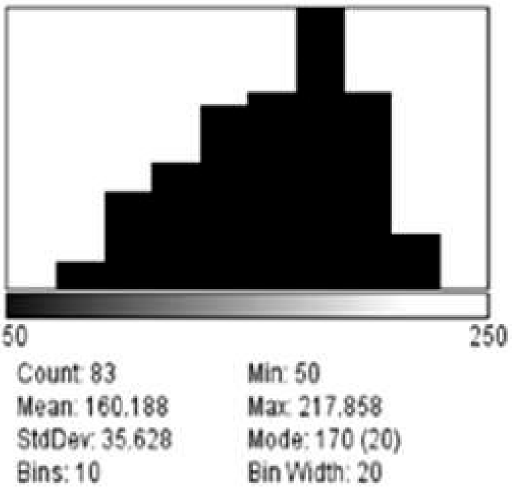

Size range of particles (nm)	Frequency	%
50-70	0	0
70-90	2	2
90-110	7	8
110-130	9	11
130-150	13	16
150-170	14	17
170-190	20	24
190-210	14	17
210-230	4	5
230-250	0	0
TOTAL	83	100

Figure S11. – Histogram and size range of the synthesized magnetic nanoparticles – Experiment 9.

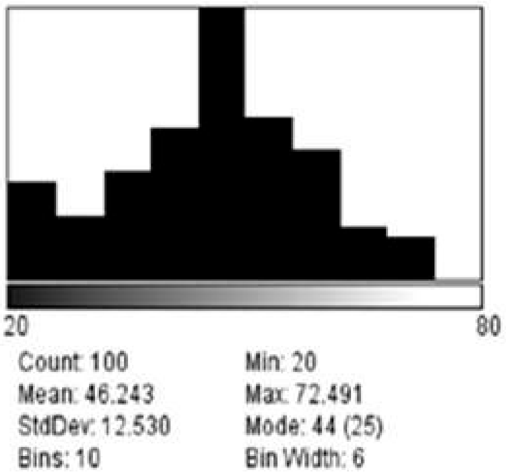

Size range of particles (nm)	Frequency	%
20-26	9	9
26-32	6	6
32-38	10	10
38-44	14	14
44-50	25	25
50-56	15	15
56-62	12	12
62-68	5	5
68-74	4	4
74-80	0	0
TOTAL	100	100

Figure S12. – Histogram and size range of the synthesized magnetic nanoparticles – Experiment 10.

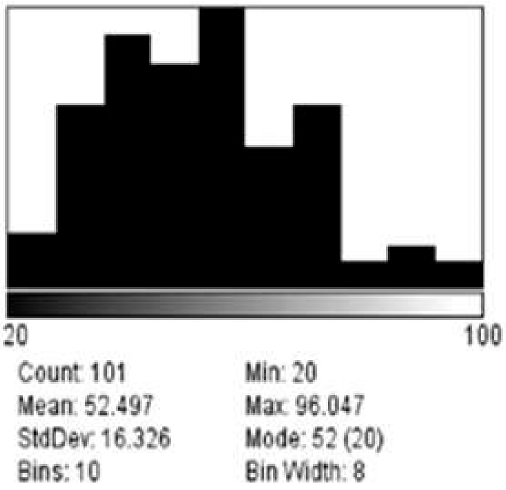

Size range of particles (nm)	Frequency	%
20-28	4	4
28-36	13	13
36-44	18	18
44-52	16	16
52-60	20	20
60-68	10	10
68-76	13	13
76-84	2	2
84-92	3	3
92-100	2	2
TOTAL	101	100

Figure S13. – Histogram and size range of the synthesized magnetic nanoparticles – Experiment 11.

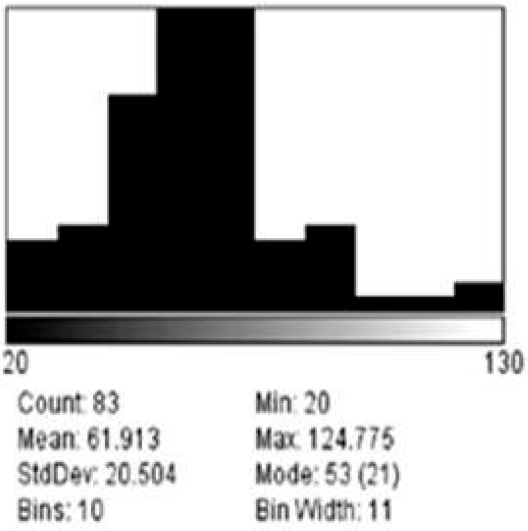

Size range of particles (nm)	Frequency	%
20-31	5	6
31-42	6	7
42-53	15	18
53-64	21	25
64-75	21	25
75-86	5	6
86-97	6	7
97-108	1	1
108-119	1	1
119-130	2	2
TOTAL	83	100

Figure S14. – Histogram and size range of the synthesized magnetic nanoparticles – Experiment 13.

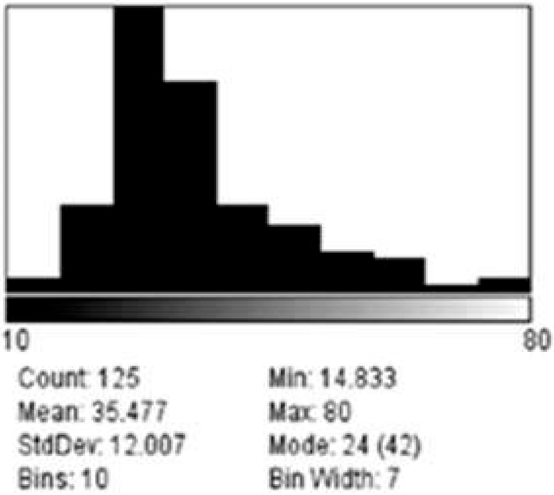

Size range of particles (nm)	Frequency	%
10-17	2	2
17-24	13	10
24-31	42	34
31-38	31	25
38-45	13	10
45-52	10	8
52-59	6	5
59-66	5	4
66-73	1	1
73-80	2	2
TOTAL	125	100

Figure S15. – Histogram and size range of the synthesized magnetic nanoparticles – Experiments 12 and 14.

C – Economic and Technological Viability

1-. Size of the Plant

To determine the size of the plant a preliminary study on the market demand must be done.

i) Market study

Nanotechnology is having a notable growth in recent years due to the potential for economic development and for the improvement of the quality of life. This is due to the great variety of applications in fields as, for example, materials, information and communication technologies, transportation, sciences of life and health and consumer and production goods.

Currently, nanotechnology is part of one of the 6 technologies essential enablers of the European Union, a key element in European industrial policy. According to the just ended "Framework Program for Research and Innovation of the Europe", Horizon 2020, it is expected that by 2020 nanotechnologies will be fully integrated into daily life, providing benefits for consumers in various areas such as food and health, and generating new industrial solutions.

For the estimation of demand for the product in question the graphics presented in [1] could have been considered. However, as in this case is being considered a more specific product for specific applications, and as data is available, it is more appropriate to address the demand in the fields of Nanomedicine. In fact, the great advantage of the product in question is that it may be applied into several areas of nanomedicine, not only concerning hyperthermia applications but also drug delivery, etc.

According to a study conducted by BBC Research, the global market for nanoparticles for biotechnology and pharmaceutical applications are expected to grow with a compound annual growth rate (CAGR) of 22%, where the highest growth will come from the drug transport area [4] – Figure S16.

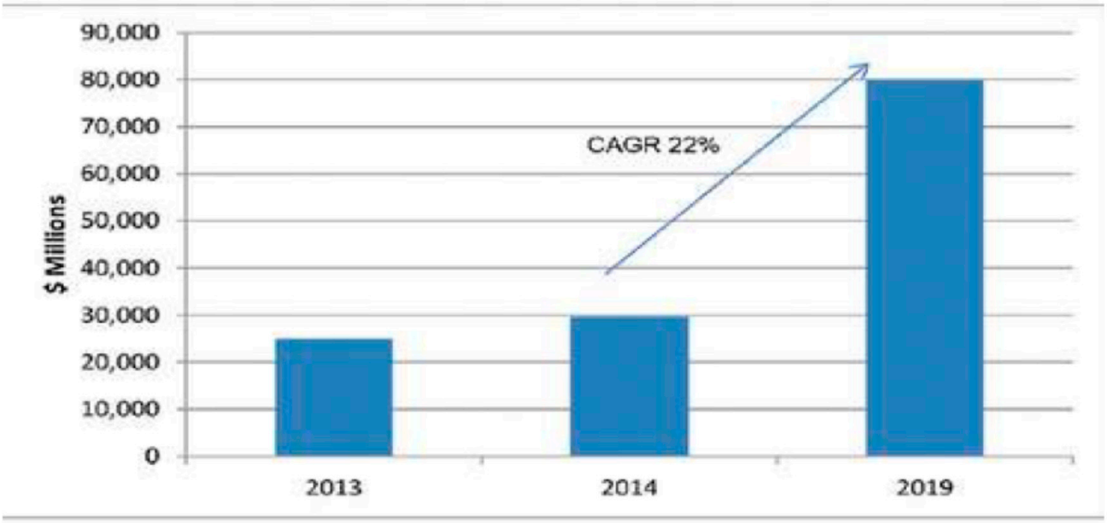

Figure S16. - Market growth for nanoparticles for biomedical applications [4].

On the other hand, considering the study carried out by the European Commission, within the scope of health, it is expected that the global market for nanomedicine products (nanopharmaceuticals and nanodiagnostic) will grow from 163 billion euros in 2013 to more than 400 thousand million in 2019, with a CAGR of more than 16% [6] – Figure S17.

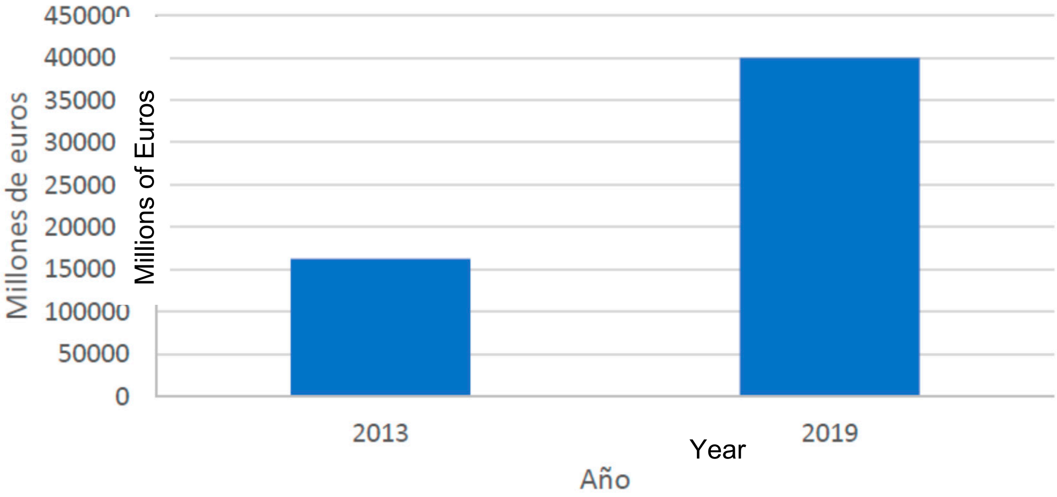

Figure S17. - Nanomedicine market growth rate until 2019 [2].

A subsequent study by BBC Research of the global market for nanotechnology, it is established that this should reach $ 90.5 billion by 2021 from $ 39.2 billion in 2016 to a CAGR of 18.2%, from 2016 to 2021 [7] – Figure S18.

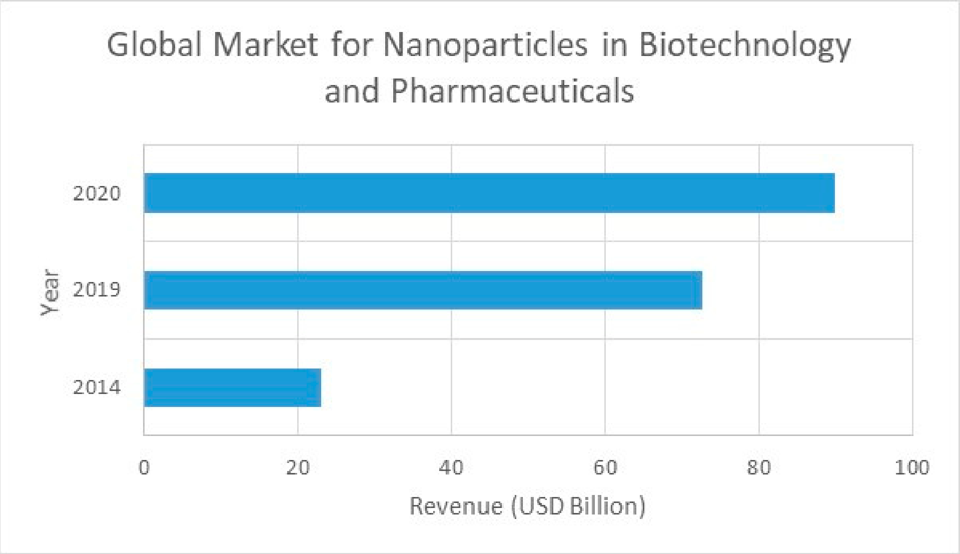

Figure S18. - Another study of the nanomedicine market until 2020 [3].

The estimation of the cost of magnetic nanoparticles, depends on characteristics such as size, shape, purity, method of synthesis, and different coatings they may have. The nanoparticles that are intended to be produced in this project will have a cubic morphology, a mean size between 20-30 nm. However, they will lack coating.

Taking into account these considerations, it will be assumed that the plant has sufficient capacity to meet 0.1% of world demand. As worldwide demand, an average value of the three studies previously mentioned will be considered, giving a result of € 203,200,000,000 for 2020. Therefore, the value generated by the plant is assumed to be € 203,200,000. The price of nanoparticles is estimated between € 10,000 / kg and € 60,000 / kg for large-scale productions and for biomedical applications. For nanoparticles synthesized in this plant, with the characteristics previously described, the price will be € 30,000 / kg, hence, the annual production is equivalent to 6,773 kg / year.

ii) Minimum production capacity

The minimum production capacity for a profitable process is always an important value to determine. This will be given by the interception of the functions of the total costs and total income *versus* production volume.

ii1) Income

The total income, due to selling the magnetic particles will be:

$$V = q * P \qquad V\left(€/_{year}\right) = 6\,773\,\frac{kg}{year} * 29\,030\,\frac{€}{kg} = 203\,200\,000€/year \qquad \text{(S1)}$$

where V is the total income, q the maximum capacity of the plant and P the price of the particles.

ii2) Costs

To determine a first estimate of the costs, the following formula is considered [2]:

$$C = M_1 + M_5 + 1.5M_2 + 0.3I \qquad \text{(S2)}$$

where C is the total cost, M_1 the raw material costs, M_5 the general services costs, M_2 the man labor costs and I the immobilized costs.

Raw Materials

The raw materials will be Iron acetylacetonate (III), Oleic acid, 4-Biphenylcarboxylic acid, Benzyl ether, Toluene, Hexane and Nitrogen. In the process 29.25 kmol of magnetite are produced per year.

For Iron acetylacetonate (III) the consumption will be 31 025 kg/year, and assuming a cost of about 128 €/kg, the total cost will be 3 971 215 €/year.

The Benzyl ether will be supplied at the price of 17.30 €/kg, and as 466 051 kg/year are needed, this represents a total cost of 8 062 682 €/year.

17571.44 kg/year of 4-Biphenylcarboxylic acid are needed, which represent a cost of 6 729 861 €/year (383 kg/year is the price of acquisition of this raw material).

In what Oleic acid is concerned, 61 980.28 kg/year are required, and if this amount is multiplied by its price (45.39 €/kg) its costs will be obtained (2 813 285 €/year).

Toluene is required at a quantity of 903 066.67 liters/year, which at a price of 10.25 €/L, gives a total cost of 9 256 433 €/year.

A cost of 6 411 773 €/year is due to the use of Hexane, as 225 766.67 liters/year are required at a cost of 28.40€/L.

At a price of 35.50 €/liter, are required 225 766.67 L/year of Chloroform, which implies a total cost of 8 014 717€/year

A quantity of 801 600 L/year of Nitrogen are required, which at a price of 3.79 €/L gives a cost of 3 038 064 €/year.

The total raw materials cost (M_1) will be 48 298 030 €/year.

General Services

In general services are considered costs such as electricity, steam, compressed air, etc. This cost usually is calculated as 10–20% of the total costs. A mean value of 15% was assumed ($M_S = 0.15$ C).

Direct Manpower (M_Z)

The required manpower will be at this stage evaluated by the Andres method [2]:

$$\frac{Hh}{Tm*op} = 61.33 * q^{-0.82} \quad \text{(S3)}$$

where *Hh* is the required manpower per hour, *Tm* is total production (tonne/year), *op* the number of process sections of the plant, and *q* the maximum capacity of the plant (tonne/day).

The plant will work semi-continuously during the year, 8 hr per day and 5 days per week, 48 weeks per year. The total number of process sectors is 4.

The total number of direct manpower is 21 workers, which corresponds to a total cost of M_2 = 956,210 €/year.

Immobilized Capital

The immobilized capital is composed by three different types of costs: the fixed active (I_A), the previous studies (I_B) and the start-up costs (I_C).

$$\boldsymbol{I = I_A + I_B + I_C} \quad \text{(S4)}$$

Its total is 367 516 730 €, according to the values of the different types of costs, detailed in the next sections.

Fixed active costs

This type of costs are the enterprise goods that may not be sell in the short time and that are not destined for sale: e.g. instrumentation, office material, etc. For its estimation the "giro coefficient method" was used [2]

$$g = \frac{V}{I_A} \quad \text{(S5)}$$

where V is the sales income and g a coefficient that changes according to the type of factory. For chemical industries it has the general value of 0.97.

In this case, I_A is equal to 209 484 536€.

Preliminary studies

The costs associated with the preliminary studies, as an industry of improved products and low-production rate is being considered, is estimated as being 35% of the total immobilized ($I_B = 0.35$ I).

Start-up costs

This is a value that also depends on the immobilized, and the fixed active costs. For this case it corresponds to 8% of the total immobilized capital (I_C = 0.08 I).

Total Costs

Applying all the data, as previously justified, a total cost of 182 140 024€/year is obtained.

Fixed Costs

Fixed costs are independent of production, so they do not vary with the quantity produced. The fixed costs of the plant correspond to 10-20% of total production costs (Vian Ortuño, 1991).

An average value of 15% will be assumed, thus Fixed Costs (FC) = 0.15 Total Costs (C). In this case the Fixed Costs are 27 321 004 €/year.

Plot costs/income vs production capacity

By plotting the curves total costs/incomes *versus* total capacity (Figure S19), the interception gives the minimum production for a profitable plant. For this case it corresponds to 3,850 kg/year.

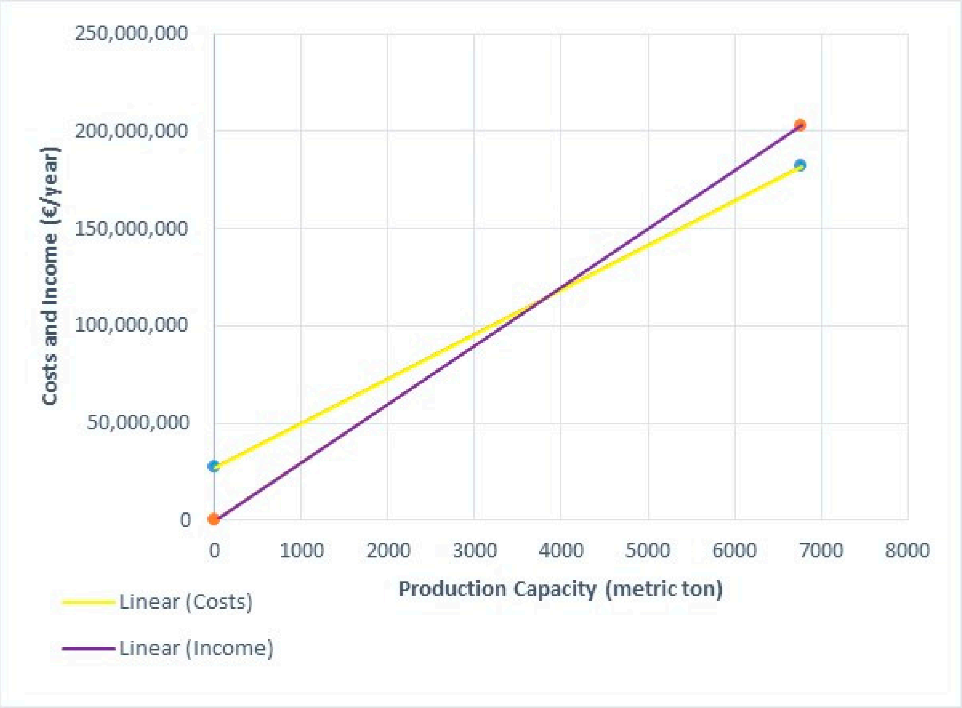

Figure S19. - Total costs/incomes *versus* total capacity.

2–. Production Cost

The total Production cost will be the sum of the Fabrication Cost (M) and the Management Cost (G)

2.1. –Fabrication Cost

(a) Cost of Raw Materials

In Table S1 are presented the Costs of Raw materials.

Table S1. –Costs of Raw Materials.

Raw Material	Price (€/kg)	Quantity (kg/year)	Cost (€/year)
Iron acetylacetonate (III)	128	31,025	3,971,215
Benzyl ether	17.30	466,051	8,062,682
4-Biphenylcarboxylic acid	383.00	17,571.44	6,729,861
Oleic acid	45.39	61,980.28	2,813,285
Toluene	10.25	903,066.67	9,256,433
Hexane	28.40	225,766.67	6,411,773
Chloroform	35.50	225 766.67	8,014,717
Nitrogen	3.79 €/l	801,600 (l/year)	3,038,064
Total: 48,298,030 €/year			

It is important to notice that it is foreseen a recycling of the raw materials that will represent a total saving of 16,543,344 €/year. This saving should be considered as an income in raw materials, and thus the real total raw materials cost would be 31 754 686 €/year. Nonetheless, as a conservative value, the maximum cost value of the raw materials in the case when the recycling process sector is not operative, will be considered.

(b) Direct Human Labor

In Table S2 are presented the labor costs for the direct human labor.

Table S2. –Direct human labor costs.

Job	Number of Workers	Annual Salary (€)	Extra Annual (incl. social security) (€)	Total (€)
**Mechanic**	5	12,862.70	8,354.24	106,084.70
**Plumber/Builder**	5	12,862.70	8,354.24	106,084.70
**Electrical Technician**	5	12,862.70	8,354.24	106,084.70
**Laboratory Technician**	5	15,258.07	12,153.64	137,058.55
**Operator Zone 1**	20	12,341.34	6,386.54	374,557.60

(c) Indirect Human Labor

In Table S3 are presented the labor costs for the indirect human labor.

Table S3. –Labor costs for indirect human labor.

Job	Number of Workers	Annual Salary (€)	Extra Annual (incl. social security) (€)	Total (€)
**Cleaning Personnel**	5	12,181.34	6,878.40	95,298.70
**Security**	2	12,337.34	6,878.40	38,431.48
**Maintenance**	1	12,337.83	6,878.40	19,216.23
**Nurse**	5	12,181.34	6,878.40	95,298.70

(d) General Services

In this section are included costs such as refrigeration, etc. this cost cab ne estimated to be 20% of the total production costs or 70% of the total costs of direct human labor. The latter was preferred, which makes a total of 580,909 €/year.

(e) Supplies

In this section are considered the items that are not included in the manufacturing process, mainly disposable material, etc. It is usually estimated to be valuing between 0.5% and 2% of the total immobilized, depending on the complexity of the plant. In this case it is assumed to be 1%, which corresponds to 80,096 €/year.

(f) Maintenance

In this section are included the periodic maintenance of instrumentation and equipment. For this specific application it usually corresponds to 7% of the fixed capital, and thus, in this case, 243,179 €/year.

(g) Laboratory

For the laboratory work is usually assumed 10-35% of the direct human labor. For this example, 20% will be assumed as the correct value. In this case it represents 165,974 € /year.

(h) Chief Personnel

In Table S4 are presented the chief personnel costs.

Table S4. – Chief Personnel labor costs.

Job	Number of Workers	Annual Salary (€)	Extra Annual (incl. social security) (€)	Total (€)
**Managing Director**	1	18,571.32	16,593.38	32,682.49
**Process Engineering**	1	15,258.07	12,153.64	27,411.71
**Environmental and Safety Engineering**	1	15,258.07	12,153.64	27,411.71
**IC Technician**	5	15,258.07	12,153.64	137,058.55

(i) Amortization

This section takes into account the life span of the equipment. It was taken as life span the usual figures concerning each type of equipment. Therefore, this cost is estimated as 82,704 €/year.

(j) Taxes

This section takes into consideration taxes related to the chemical plant (general worldwide), not including the taxes due to profits. It usually is estimated as 1% of the Immobilized. Hence, in this case is estimated to be 80,096 €/year.

(k) Insurance

In this section the insurance of the plant (equipment, facilities, etc.) is considered. It is usually 1% of the Immobilized, so, it is estimated to represent a cost of 80,096 €/year.

(l) Total Cost of Fabrication

TOTAL FABRICATION COSTS: 50,332,893.00 €/year.

2.1.2–Management Costs

(a) Commercial Costs

The commercial costs are estimated to be 20% of the total fabrication costs, thus representing a total value of 10,068,379.00 €/year.

(b) Financial Costs

In this item the amount of money that could be earned is considered, if instead of investing it in each plant, it would be invested in a fixed deposit in a bank or other competing alternatives. For the application in question and as several alternative opportunities are usually available, therefore, a value of 20% was assumed giving a total cost of 5,809,661 €/year.

(c) Management

In this section the administrative management costs are considered or alternatively (depending on the type and dimensions of the plant) the cost of a full sector of the plant dedicated to management. For the application under study a simpler sector of management is required, therefore in Table S5 are presented the costs related to this sector that give a total of 131,826.49 €/year.

Table S5. –Management Personnel labor costs.

Job	Number of Workers	Annual Salary (€)	Extra Annual (incl. social security) (€)	Total (€)
Administrative	1	14,202.87	10,538.61	24,741.48
Recepcionist	1	12,337.83	6,878.40	19,216.23
Commercial	1	12,364.34	7,660.05	20,024.39
Secretary	1	12,337.34	6,878.40	19,215.74
Manager	1	15,258.07	12,153.64	27,411.71
Accountant	1	12,862.70	8,354.24	21,216.94

(d) Research, Development and Technical Services

The costs on R&D may be estimated as 3–4% of the Immobilized, depending on the level of novelty of the product. For the application in question, due to the several research already done, 3% was considered as more appropriate, and thus the R&D costs for this type of plant is 240,286 €/year. Considering Technical Services the costs of technical services will be calculated as 1% of the total selling income due to the high price of the product (Total cost of 2,032,000 €/year).

(e) Total Cost of Management

TOTAL COST OF MANAGEMENT: 18,386,548 €/year.

2.1.3–Total Cost of Production

Total Cost of Production = Total Cost of Fabrication (2.1.1) + Total Cost of Management (2.1.2), thus:

TOTAL COST OF PRODUCTION: 69,250,424 €/year.

3–. Invested Capital

The Invested Capital is the sum of two parts: the immobilized and the working capital

3.1. –Fixed Capital

This is the amount of invested money that will not be recovered, and is calculated as the sum of parts (a) to (h) that are detailed in what follows. It gives a total of 3,473,984 €/year.

(a) Equipment and Instrumentation

The total cost of equipment reaches the 827,043 €/year.

(b) Assembly and Start-up

Usually the costs for assembly and startup of the devices and equipment are determined as a percentage of the total equipment cost, therefore will be a total of 473,602 €/year.

(c) Tubing and valves

The cost of tubing and valves is estimated as 30-60% of the total equipment costs, and hence for this plant it will be considered a 45%, which gives total of 372,270 €/year.

(d) Measuring and Control Devices

Costs of this section are usually estimated between 15–30% of the total equipment costs, depending on the level of control required for the plant. For this application it was considered 20%, and therefore the total costs are 165,409 €/year.

(e) Thermal Isolation

The cost for the thermal isolation will be considered to be 7% of the total equipment costs as thermal isolation in this application is a high requirement (higher temperatures must be maintained and operated). This gives a total of 57,893 €/year.

(f) Electrical Installation

The cost for the electrical installation, usually corresponds to a percentage of the total equipment costs. For this case it is calculated as 15% of the equipment cost, as moderate equipment and area is covered (124,057 €/year).

(g) Land Property and Buildings

This is calculated by adding the cost of the land as a percentage of the total equipment costs. For this case it is assumed 25% of the equipment costs, that added to the cost of the land (916,132 €) gives a total of 1,122,893 €/year.

(h) Auxiliary Facilities

For this type of cost, usually it is estimated a value of 40% of the total equipment cost, that gives a total value of 330,817 €/year.

3.2.–. Other Costs

(a) Design and Engineering

This part contemplates all the design and engineering works done for the construction of the plant. Usually it is estimated as 10.4% (including VAT) of the total Fixed Capital, and therefore will be a total of 361,923 €/year.

(b) Contract of Works

It is supposed to be 6% of the fixed capital, thus will be a total of 208,439 €/year.

(c) Contingency

In this section are included all the unexpected extra costs that may appear. It is usually estimated as being a percentage of the Fixed Capital, depending on the level of estimative costs that has been made and the unexpected problems foreseen. For the case under analysis 15% will be considered as a correct amount (total of 521,098 €/year).

(d) Preliminary Research, Studies and Startup

The costs related to preliminary research studies and startup may be calculated as 35% and 8% of the total immobilized, respectively. The total gives 3,444,106.88 €/year.

Fixed Immobilized Capital

The total Fixed Immobilized capital is calculated as the sum of the Fixed Capital and the Other Costs. In this case it gives a total of 8,009,550.88€.

4–. Working Capital

This is the part of the Capital that may be recovered.

4.1. –Raw Materials and Auxiliaries

To compute this cost it may be applied

$$M'_1 * \left(\frac{q}{12}\right) \quad \text{(S6)}$$

where M'_1 is the cost of raw materials by unit of product and q the annual production quantity. This gives a final cost of 4,024,835 €/year for this section of the costs.

4.2. –Materials in Manufacturing

This cost may be computed with the knowledge of the manufacturing cycle. In this case corresponds to 0.033 months and therefore the associated costs will be 69,938 €/year.

4.3. –Reserve of the Manufactured Product in the Warehouse

The costs corresponding to this section are computed as 4,238,656 €/year, as they are obtained as the fabrication cost per month.

4.4. –Sales Pending Collection

Corresponds to the credit that is given to buyers. In this case it corresponds to 8,466,667 €/year –calculated as the income (of the selling of the product) per each 15 days.

4.5. –Available in ATM's and Banks for Immediate Payments

It corresponds to 4,238,656 €/year. They are estimated in a similar way as the reserve of the manufactured product in the warehouse.

4.6. –Calculation of Working Capital

TOTAL WORKING CAPITAL: 21,038,753 €/year

5–. Total Invested Capital

TOTAL INVESTED CAPITAL = WORKING CAPITAL + IMMOBILIZED
TOTAL INVESTED CAPITAL: 29,048,303 €/year

6. –Total Sell Income

This was already calculated previously and is equal to 203,200,000 €/year.

7–. Profitability

7.1. –Gross Annual Profit

This is computed as the difference between Costs and Sells, and in this case gives a value of 133,949,576 €/year.

7.2. –Gross Annual Profitability

Is computed as the Gross Annual Profit divided by the Invested Capital. In this case it gives a value of 4.6 times the invested capital.

7.3–. Gross Annual Profit over Costs

Is computed as the Gross Annual Profit divided by the Costs and multiplied by 100. In this case it gives a value of 193%.

7.4. –Net Annual Profit

Is equal to the Gross Annual Profit minus the Taxes. In this case as the Society Tax is 25% in Spain, a total Net Annual Profit of 100,462,182 €/year is obtained.

7.5–. Net Annual Profitability

In this case study it gives a value of 3.4 times the invested capital.

7.6–. Gross Annual Profit over Costs

Is computed as the Net Annual Profit divided by the Costs and multiplied by 100 giving a value of 145%.

C – Process Engineering.

Mass and Heat Balances – Streams

In Table S6 are presented the details of the streams of the processing flowsheet shown in Figure 4 of the main text. The details reflect all the results obtained from mass and heat balances and the application of the processing units.

Table S6. – Streams of the Plant and their characteristics.

Streams	1	2	3	4	5	6	7	8	9	10	11	12	13	14	15	16	17	18	19
**Compounds**	**Mass flow (kg/day)**																		
**Iron (III) acetylacetonate**	93.32				93.32	31.11	31.11	31.11											
**Oleic acid**		166.93			166.93	55.64	55.64	55.64				3.90	3.90	3.90	3.90	3.90	3.90		
**4-Biphenylcarboxylic acid**			52.58		52.58	17.53	17.53	17.53				17.53	17.53	17.53	17.53	17.53	17.53		
**Benzyl ether**				1366.98	1366.98	455.66	455.66	455.66				455.66	455.66	455.66	455.66	455.66	455.66		
**Nitrogen**									0.50	0.50	0.50								
**Magnetite**												6.76	6.76	6.76	6.76	6.76	6.76		
**Subproducts**												76.10	76.10	76.10	76.10	76.10	76.10		
**Hexane**																		430.47	
**Toluene**																			2279.17
**Ethylene glycol**																			
**Chloroform**																			
**T (K)**	298.00	298.00	298.00	298.00	298.00	298.00	298.00	298.00	298.00	298.00	298.00	563.00	563.00	563.00	298.00	298.00	298.00	298.00	298.00
**P (kPa)**	101.30	101.30	101.30	101.30	101.30	101.30	101.30	101.30	101.30	101.30	101.30	101.30	101.30	101.30	101.30	101.30	101.30	101.30	101.30
**Enthalpy (kJ/day)**	-3.47E+05	-3.97E+05	1.56E+04	1.33E+05	-5.96E+05	-1.99E+05	-1.99E+05	-1.99E+05				5.37E+04	5.37E+04	5.37E+04	-1.54E+04	-1.54E+04	-1.54E+04	-9,21E+05	4.29E+06
**Total Mass Flow (kg/day)**	93.32	166.93	52.88	1366.98	1679.80	559.93	559.93	559.93	0.5	0.5	0.5	559.93	559.93	559.93	559.93	559.93	559.93	430.47	2279.17
**Total Molar Flow (kmol/day)**	0.26	0.59	0.27	6.89	8.02	2.67	2.67	2.67	0.02	0.02	0.02	2.52	2.52	2.52	2.52	2.52	2.52	5.01	24.77

Streams / Compounds	20	21	22	23	24	25	26	27	28	29	30	31	32	33	34	35	36	37
**Iron (III) acetylacetonate**																		
**Oleic acid**	11.69																11.69	
**4-Biphenylcarboxylic acid**	52.58	52.58	52.58	52.58														
**Benzyl ether**	1366.98	1366.98	1366.98	1366.98														
**Nitrogen**																		
**Magnetite**	20.28																	20.28
**Subproducts**	228.29	228.29	228.29		228.29	228.29		228.29	228.29		4.57	223.72	223.72	0.04	223.68			
**Hexane**	430.47	430.47	430.47	0.22	430.25	430.25	0.22	430.04	430.04		430.04							
**Toluene**	2279.17	2279.17	2279.17	1.14	2278.03	2278.03	2278.03											
**Ethylene glycol**										329.16		329.16	329.16	329.10	0.07			
**Chloroform**																979.23	979.23	
**T (K)**	298.00	298.00	379.60	530.00	377.20	366.00	383.30	337.60	298.00	298.00	298.00	563.00	357.80	470.20	334.00	298.00	298.00	298.00
**P (kPa)**	101.30	101.30	101.30	101.30	101.30	101.30	101.30	101.30	101.30	101.30	101.30	101.30	101.30	101.30	101.30	101.30	101.30	101.30
**Enthalpy (kJ/day)**	3.33E+06	3.45E+06	4.10E+06	7.02E+05	4.83E+06	3.68E+06	4.62E+06	-6.57E+05	-9.89E+05	-2.08E+06	-9.22E+05	-2.15E+06	-2.07E+06	-1.95E+06	7.35E+04	-8.48E+05	-8.76E+05	-7,55E+04
**Total Mass Flow (kg/day)**	4389.44	4357.48	4357.48	1420.91	2936.57	2936.57	2278.25	658.32	658.32	329.16	434.60	552.88	552.88	329.14	223.74	979.23	990.91	20.28
**Total Molar Flow (kmol/day)**	37.34	37.22	37.22	7.17	30.04	30.04	24.76	5.28	5.28	0.40	5.01	5.58	5.58	5.31	0.27	8.23	8.28	0.09

1. Augusto, P.A.; Castelo-Grande, T.; Vargas, D.; Pascual, A.; Hernández, L.; Estevez, A.M.; Barbosa, D. Upscale Design, Process Development, and Economic Analysis of Industrial Plants for Nanomagnetic Particle Production for Environmental and Biomedical Use. *Materials* **2020**, *13*, 2477, doi:10.3390/ma13112477.
2. Vian-Ortuño, Á. El Pronóstico Económico en Química Industrial; EUDEMA, S.A.: Madrid, Spain, 1991.
3. Sinnot, R. Diseño en Ingeniería Química; Reverté: Barcelona, Spain, 2012.
4. James, K.; Highsmith, J.; Evers, P. Drug Development and Delivery; Available online: https://drug-dev.com/ (accessed on 28 Janruary 2021).
5. Gómez Roca, A. *Preparación de Nanopartículas Magnéticas Uniformes y de Alta Cristalinidad para Biomedicina*; Instituto de Ciencia de Materiales de Madrid, Departamento de Química Física I, Universidad Complutense de Madrid: Madrid, Spain, 2009.
6. ICON. Evolution of Nanotechnology in Spain, 2018, Fundación Española para la Ciencia y la Tecnología. Available online: https://icono.fecyt.es/ (accessed on 28 Janruary 2021).
7. The Maturing Nanotechnology Market: Products and Applications. Available online: https://www.bccresearch.com/market-research/nanotechnology/nanotechnology-market-products-applications-report.html (accessed on 28 Janruary 2021).
